# DNA transfer between two different species mediated by heterologous cell fusion in *Clostridium* coculture

**DOI:** 10.1128/mbio.03133-23

**Published:** 2024-01-12

**Authors:** Kamil Charubin, John D. Hill, Eleftherios Terry Papoutsakis

**Affiliations:** 1 Department of Chemical and Biomolecular Engineering, The Delaware Biotechnology Institute, University of Delaware, Newark, Delaware, USA; University of Massachusetts Amherst, Amherst, Massachusetts, USA

**Keywords:** *Clostridium ljungdahlii*, *Clostridium acetobutylicum*, syntrophy, heterologous cell fusion, DNA exchange, DNA integration, plasmid DNA

## Abstract

Prokaryotic evolution is driven by random mutations and horizontal gene transfer (HGT). HGT occurs via transformation, transduction, or conjugation. We have previously shown that in syntrophic cocultures of *Clostridium acetobutylicum* and *Clostridium ljungdahlii*, heterologous cell fusion leads to a large-scale exchange of proteins and RNA between the two organisms. Here, we present evidence that heterologous cell fusion facilitates the exchange of DNA between the two organisms. Using selective subculturing, we isolated *C. acetobutylicum* cells which acquired and integrated into their genome portions of plasmid DNA from a plasmid-carrying *C. ljungdahlii* strain. Limiting-dilution plating and DNA methylation data based on PacBio Single-Molecule Real Time (SMRT) sequencing support the existence of hybrid *C. acetobutylicum*/*C. ljungdahlii* cells. These findings expand our understanding of multi-species microbiomes, their survival strategies, and evolution.

**IMPORTANCE:**

Investigations of natural multispecies microbiomes and synthetic microbial cocultures are attracting renewed interest for their potential application in biotechnology, ecology, and medical fields. Previously, we have shown the syntrophic coculture of *C. acetobutylicum* and *C. ljungdahlii* undergoes heterologous cell-to-cell fusion, which facilitates the exchange of cytoplasmic protein and RNA between the two organisms. We now show that heterologous cell fusion between the two Clostridium organisms can facilitate the exchange of DNA. By applying selective pressures to this coculture system, we isolated clones of wild-type *C. acetobutylicum* which acquired the erythromycin resistance (erm) gene from the *C. ljungdahlii* strain carrying a plasmid with the erm gene. Single-molecule real-time sequencing revealed that the erm gene was integrated into the genome in a mosaic fashion. Our data also support the persistence of hybrid *C. acetobutylicum*/*C. ljungdahlii* cells displaying hybrid DNA-methylation patterns.

## INTRODUCTION

The evolution of bacteria and other single-cell organisms is facilitated through genetic mutations and horizontal gene transfer (1–4). In mutation-driven evolution, a cell undergos random mutagenesis. The mutations are then passed down to its daughter cells. In the horizontal gene transfer (HGT) process, cells acquire exogenous DNA transferred from other cells, often from a different species (1). The first evidence for HGT was provided in 1928, where pneumococci (in infected mice) exchanged virulence genes through the uptake of genetic DNA (1, 5). Since then, many gene transfer and genetic recombination examples have been demonstrated in the laboratory while genetic exchanges occurring in nature are believed to be widespread (1, 4). The most-studied mechanisms of HGT between bacteria include transformation, transduction, and conjugation. Additional mechanisms of HGT have been identified including nanotube formation (6) and potential transfer via extracellular vesicles (7, 8).

In transformation, competent cells take up genomic or plasmid DNA from their environment (1, 4). Natural competence involves 20–50 proteins (1) and has been documented in more than 80 species (3). Artificial competence can be developed in the laboratory through chemical treatment (4). Transformation does not require cell-to-cell contact, as competent cells can take up foreign DNA from their environment. In transduction, DNA exchange between cells is carried out by a bacteriophage (4). Transduction transfer frequencies vary greatly depending on the organism and phage pair and are of the order of 10^−3^ to 10^−9^ per plaque-forming unit (9). This occurs when a portion of the chromosomal DNA of a parent organism is accidentally packaged when a latent prophage excises from the genome. The phage containing the bacterial DNA can interact with and infect other cells, thus transferring the packaged DNA (4). Transduction does not require any cell-to-cell contact between cells either. Conjugation requires two bacterial cells to interact physically to exchange DNA (1) and has been characterized in most detail in Gram-negative bacteria***,*** such as *Escherichia coli*. Mating requires the cocultivation of donor and recipient cells on a solid surface. The conjugative machinery is encoded on a conjugative plasmid or integrative and conjugative elements (ICEs), a subset of which includes conjugative transposons (1, 2, 10). In Gram-negative bacteria, the cell-to-cell contact and DNA exchange between the donor and the recipient cell are typically mediated by a type IV secretion system (T4SS) which utilizes a pilus (the mating-pair formation apparatus) (1). Some Gram-positive bacteria, like *Staphylococcus* and *Enterococcus* species*,* use systems homologous to the T4SS of Gram-negative bacteria (2). In Gram-positive bacteria, conjugation frequencies range from 10^−3^ to 10^−6^ depending on the plasmid and the mating pair (11). Conjugative plasmids have been identified in *Clostridium* species, an example of which includes the well-studied conjugative pCW3-like plasmids in *Clostridium perfringens* (12). Transfer of conjugative transposons from the Tn916/Tn1545 family into several *Clostridium* species from *E. coli* and *Enterococcus faecalis* has been reported, including *Clostridium tetani*, *Clostridium acetobutylicum*, and *Clostridium beijerinckii*, with transfer between members of the genus also reported (13–15). It was reported that a Tn1545 self-mobilizing transposon, that conferred both tetracycline and erythromycin resistance, was transferred from *C. beijerinckii* to *Eubacterium cellulosolvens* (16). However, no native conjugation systems have been found in either the autotrophic acetogen *Clostridium ljungdahlii* or the heterotrophic solventogen *C. acetobutylicum* (2). There is another form of conjugation, known as distributive conjugal transfer (DCT), which was more recently discovered and largely studied in mycobacteria (17, 18). Reported frequencies for distributed conjugal transfer (Table S3 of reference 18) range from 2 × 10^−4^ to <10^−8^ per donor cell, and per recipient cell from 2 × 10^−5^ to <10^−8^.

We have reported (19) that, in the syntrophic coculture of *C. acetobutylicum* and *C. ljungdahlii*, there is unexpected heterologous cell fusion between the two organisms, whereby *C. ljungdahlii* fuses with *C. acetobutylicum* polarly. These fusion events lead first to transient exchange of cellular material as documented by heterologous fluorescent protein and RNA gradients leading to uniform distribution of heterologous fluorescent material, thus demonstrating large-scale exchange of cellular proteins and RNA. Here, we provide evidence that this heterologous cell fusion leads also to plasmid and possibly chromosomal DNA exchange between the two organisms. Plasmid DNA (p100ptaHalo) was successfully transferred from the *C. ljungdahlii*-ptaHalo strain—expressing the HaloTag protein (20)—to wild-type (WT) *C. acetobutylicum*. Plasmid or other DNA transfer between these two organisms cannot take place through natural competency, as there is no evidence that either *C. acetobutylicum* or *C. ljungdahlii* can become naturally competent (2, 3), and both are difficult to transform. Significantly, the restriction-modification (RM) system of *C. acetobutylicum* prevents transformation with *C. ljungdahlii*-propagated plasmids. Since neither organism possesses identifiable conjugation machinery, the DNA exchange we report here likely occurred through the newly identified heterologous cell-to-cell fusion (19). We also provide evidence for a new form of interspecies-mediated *C. acetobutylicum* acquisition of antibiotic (erythromycin) resistance.

## RESULTS

### Coculture-mediated transfer of plasmid DNA from *C. ljungdahlii*-ptaHalo strain to WT *C. acetobutylicum*

**Fig 1:**
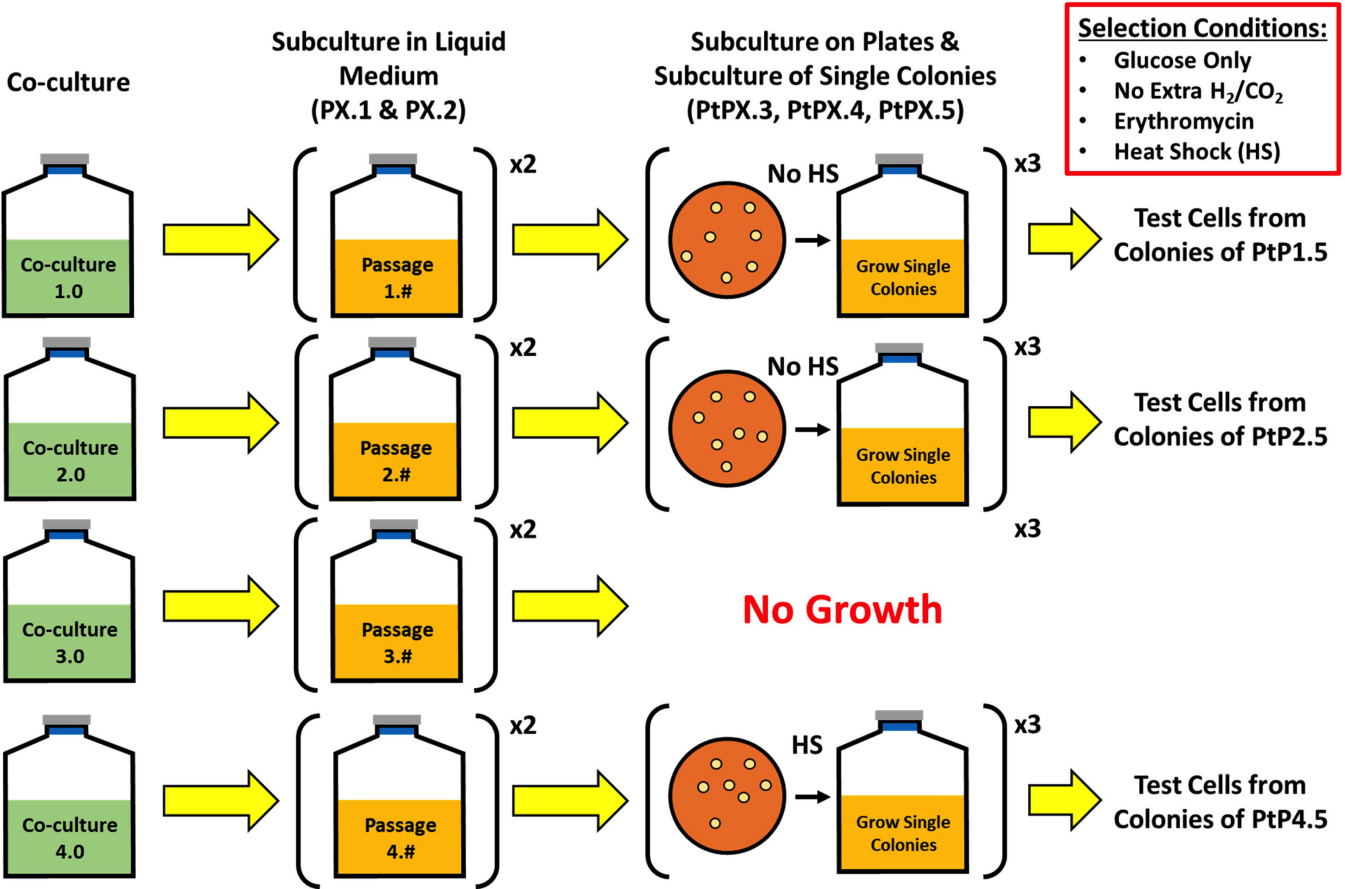
Summary of the selection procedure for isolating *C. acetobutylicum* strains, which have acquired p100ptaHalo plasmid DNA from *C. ljungdahlii*-ptaHalo cells in the coculture. Selection started with four parent cocultures (selection lines/lineages) on a growth medium containing 80 g/L glucose and 5 g/L fructose but no erythromycin (Erm). Subculture passages 1 and 2 (PX.1 and PX.2; X represents the starting biological replicate of cocultures 1 through 4) were enrichment passages in selective liquid media containing glucose as the sole carbon source and Erm. Samples from the enriched cultures PX.2 were plated on selective plates (Erm, glucose) to identify and isolate single colonies of PtPX.3 strains. Selection subculture P3.2 did not survive on plates and was abandoned. Selection subcultures P1.3 and P2.3 (and all subsequent subcultures) could not survive heat shock, indicating the lack of sporulation ability, and were subcultured without heat shock for further analysis. Selective subculture P4.3 (and all subsequent subcultures) survived the heat shock, indicating the presence of *C. acetobutylicum* cells capable of sporulation. Each subculture in the liquid selection medium is represented as PX.#, where ”X” represents the parent coculture, while the “#” represents each subsequent subculture (passage). Subcultures on selective plates are indicated as PtPX.#.

To examine whether DNA is transferred between *C. ljungdahlii* and *C. acetobutylicum* during heterologous cell fusion events, four biological replicates of parent cocultures of the *C. ljungdahlii*-ptaHalo strain, carrying the p100ptaHalo plasmid, and WT *C. acetobutylicum* were set up (Fig. 1). p100ptaHalo carries the HaloTag gene and the erythromycin (Erm) resistance gene, *erm*, and can be propagated and expressed in both organisms (20). Parent cocultures were set up in a non-selective coculture medium containing glucose and fructose but no antibiotic to allow heterologous cell fusion (19, 21). The process involved subculturing in a selective liquid medium for the first two passages, followed by plating on selective agar plates, and liquid subculturing of colonies from plates. A selective medium was chosen for subsequent passages to select for *C. acetobutylicum* cells that may have acquired p100ptaHalo from *C. ljungdahlii*-ptaHalo while eliminating *C. ljungdahlii*-ptaHalo cells over time. Liquid subcultures were carried out in the selective Turbo CGM medium containing glucose but no fructose and supplemented with Erm while plating was performed on 2× YTG plates with Erm. *C. ljungdahlii* can grow on fructose but not on glucose, and its growth is in fact inhibited by high glucose concentrations (21). WT *C. acetobutylicum* cannot grow in the presence of Erm, which was used to eliminate WT *C. acetobutylicum* cells during the selection process. The selection procedure was designed to isolate colonies of pure *C. acetobutylicum* cells that have acquired the plasmid DNA (p100ptaHalo) or the *erm* gene.

Each subculture (passage [P]) is identified as PX.#, where the “X” represents the different starting coculture (lineage), while the “#” identifies each subsequent passage in a selective medium. Passages where plating on a selective medium was performed to isolate single colonies (starting with passage PX.3) are represented by the plate (Pt) number PtPX.# while planktonic cultures grown from these colonies are denoted PX.#. It follows that the first passage of coculture 1 in the liquid medium is represented by P1.1, while the third passage of coculture 4 on selective plates is represented by PtP4.3. After each plating (PtPX.#), several isolated colonies from sparse plates were picked and grown in a selective liquid medium (PX.#); all colonies which showed growth in culture were plated again on selective plates to continue the selection process.

To start the selection process, after 24 h of growth, 15 mL samples from each parent co-culture were washed in Turbo CGM medium (80 g/L of glucose only, no fructose) and transferred to 20 mL of the liquid selection medium. The co-culture samples were washed to remove any fructose left over from the parent co-culture growth medium. This was the 1st selection PX.1. After 25 h of incubation of these planktonic cultures, samples from each PX.1 selection subculture were transferred to fresh liquid selective medium to further enrich for plasmid-containing *C. acetobutylicum* cells (15 mL of PX.1 liquid cultures was spun down, washed, and transferred to 20 mL of fresh selection medium) After 72 h, samples from subcultures P1.2 and P2.2 were streaked onto 2×YTG selection plates (plate subculture PtPX.3) to isolate and test single colonies. The growth profiles of these cultures are shown in Fig. S1. Samples from subcultures P3.2 and P4.2 were streaked on plates after 44 h of incubation. Selection plates PtPX.3 developed colonies after 2 days, except for selection plate PtP3.3 which did not develop any colonies. Thus, the 3rd lineage was abandoned. We note then that, for lineages 1, 2, and 4, *C. acetobutylicum* survived two liquid subcultures on a selective medium with Erm, which means that *C. acetobutylicum* cells had acquired the ability to resist Erm under planktonic culture conditions. *C. ljungdahlii* cells, which cannot use glucose, would have survived based on the CO_2_ and H_2_ produced by *C. acetobutylicum*. Eight to ten colonies were picked from each successful selection plate (PtP1.3, PtP2.3, and PtP4.3) and subcultured in a liquid selective medium (P1.3, P2.3, and P4.3). Half of the selected colonies were heat shocked at 80°C for 10 min, per standard *C. acetobutylicum* culture practice, to select and grow *C. acetobutylicum* cells that have sporulated. Heat shocking kills *C. ljungdahlii* cells, as they are unable to sporulate. Colonies from plates PtP1.3 and PtP2.3 did not survive to heat shock but grew in liquid selection medium if not heat shock. Colonies from plate PtP4.3 survived the heat shock and grew in liquid selection medium. All colonies that grew in liquid medium were plated on 2xYTG selection plates (PtPX.4), and the subculture process was repeated once more (passages PtPX.5), aiming to identify *C. acetobutylicum* cells resistant to Erm. Finally, four individual colonies from plates PtP1.5, PtP2.5, and PtP4.5 were grown in a liquid selection medium (P1.5, P2.5, and P4.5), and analyzed using microscopy, flow cytometry for fluorescent HaloTag expression, metabolite analysis, and PCR assays to determine whether plasmid DNA was transferred from *C. ljungdahlii*-ptaHalo to *C. acetobutylicum* cells.

**Fig 2:**
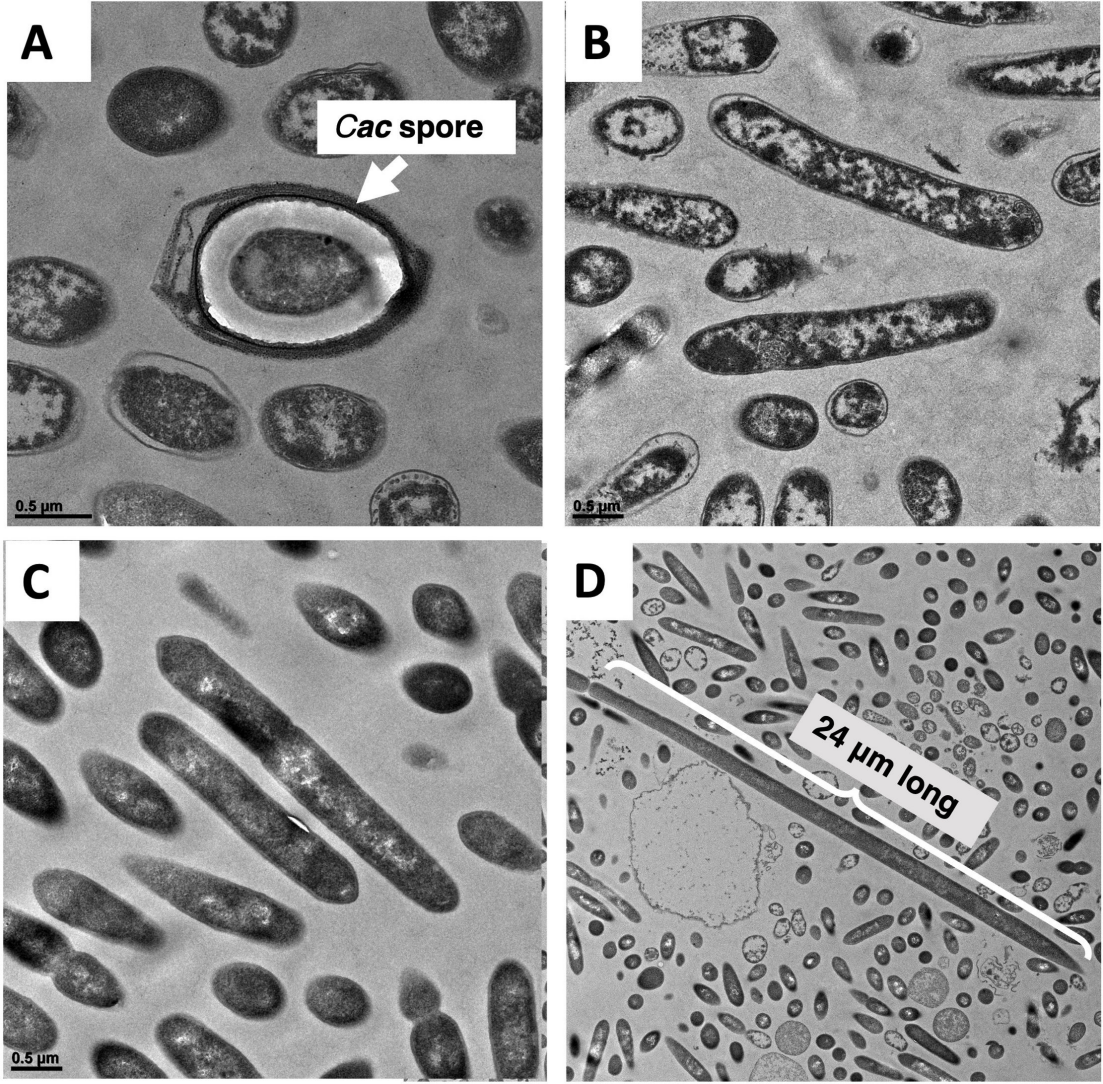
TEM imaging of cells. (**A, B**) Cells grown from colonies from a PtP4.5 plate. (**A**) A fully formed *C. acetobutylicum* spore; (**B**) cells showing formation of granulose, which are the large white and translucent regions expected of sporulating WT *C. acetobutylicum* cells. TEM imaging on PtP1.5 (**C**) and PtP2.5 (**D**) cells. (**C**) All cells had an ambiguous morphology, displaying some differentiation and granulose formation (*C. acetobutylicum*-specific phenotype), but not well defined as would be expected of pure *C. acetobutylicum* cells (compared to Fig. **S5**). (**D**) All cells had an ambiguous morphology, displaying some differentiation and granulose-like formation, and several cells were exceptionally long.

Cell phenotype was assessed based on six characteristics (Table 1): survival of heat shock, growth on glucose only, production of butanol and acetone, Erm resistance, HaloTag fluorescence, and production of isopropanol (coculture phenotype only) (21) (Table 1). Only cells grown from colonies from PtP4.5 plates showed all expected phenotypes of *C. acetobutylicum* cells that acquired Erm resistance from the *C. ljungdahlii-pta*Halo strain, except for the plasmid-specific HaloTag fluorescence (Table 1). P4.5 cells survived heat shock, grew on glucose as a substrate, were resistant to Erm, and produced butanol and acetone (Fig. S2), all of which, except for Erm resistance, are specific characteristics of WT *C. acetobutylicum* cells. Since these cells produce the characteristic solvents of *C. acetobutylicum* cells, the cells must contain these solventogenic genes, which are coded on the pSOL1 megaplasmid. Loss of pSOL1 results in an asporogenous phenotype (22), and thus the ability to withstand the heat shock further confirms the presence of pSOL1. Significantly, no isopropanol was detected in cultures of P4.5 cells despite the presence of 40 mM acetone (Fig. S2), indicating that the selection process worked at eliminating any detectable *C. ljungdahlii*-ptaHalo cells. Any small amounts of acetone are readily converted to isopropanol by *C. ljungdahlii* cells (21). Erm resistance of P4.5 cells can only be explained by the presence of the *erm* gene in the isolated cells. However, P4.5 cells did not show any red fluorescence when labeled with the red HaloTag ligand Janelia Fluor (Fig. S3). Analysis of earlier cultures from plates PtP4.3 and PtP4.4 showed that ~30% of P4.3 cells exhibited red fluorescence, but P4.4 cells did not show any red fluorescence (Fig. S3 and S4). Thus, during the 4th selective passage, cells most likely lost the ability to produce sufficient levels of HaloTag for fluorescence detection. These data show that at least part of p100ptaHalo, carrying the *erm* gene, was transferred to *C. acetobutylicum*. We also examined P4.5 cells morphologically using transmission electron microscopy (TEM). After 24 h of culture, P4.5 cells had the appearance of WT *C. acetobutylicum* cells (19): there were a few fully formed spores (Fig. 2A), and virtually all cells displayed large translucent regions in their cytoplasm (Fig. 2B) indicative of the formed granulose in preparation for spore formation. *C. ljungdahlii* cells (Fig. S5) appear only as homogeneously electron-dense (dark) vegetative cells (19), and no such cells were detected via TEM analysis.

**TABLE 1:** Phenotypic checklist of parent strains used for the starting coculture (*C. ljungdahlii-*ptaHalo and WT *C. acetobutylicum*), the expected phenotype of *C. acetobutylicum* cells which acquired the plasmid DNA (same as the *C. acetobutylicum*-ptaHalo strain), and the observed phenotype of isolated clones from plate PtP4.5

Characteristic phenotype	*C. ljungdahlii* -ptaHalo	Wild-type *C. acetobutylicum*	*C. acetobutylicum -*ptaHalo (p100ptaHalo)	Colonies from PtP4.5 Plate	Specificity
1. Heat shock survival	No	Yes	Yes	Yes	*C. acetobutylicum* specific
2. Growth on glucose	No	Yes	Yes	Yes	
3. Production of butanol and acetone	No	Yes	Yes	Yes	
4.Erythromycin resistance	Yes	No	Yes	Yes	Plasmid specific
5. HaloTag fluorescence	Yes	No	Yes	No	
6. Production of isopropanol	No	No	No	No	Co-culture specific

PCR and Single-Molecule Real Time (SMRT) (PacBio) analyses of P4.5 cells identify them as *C. acetobutylicum* cells containing both *erm* and HaloTag genes integrated into the *C. acetobutylicum* genome.

**Fig 3:**
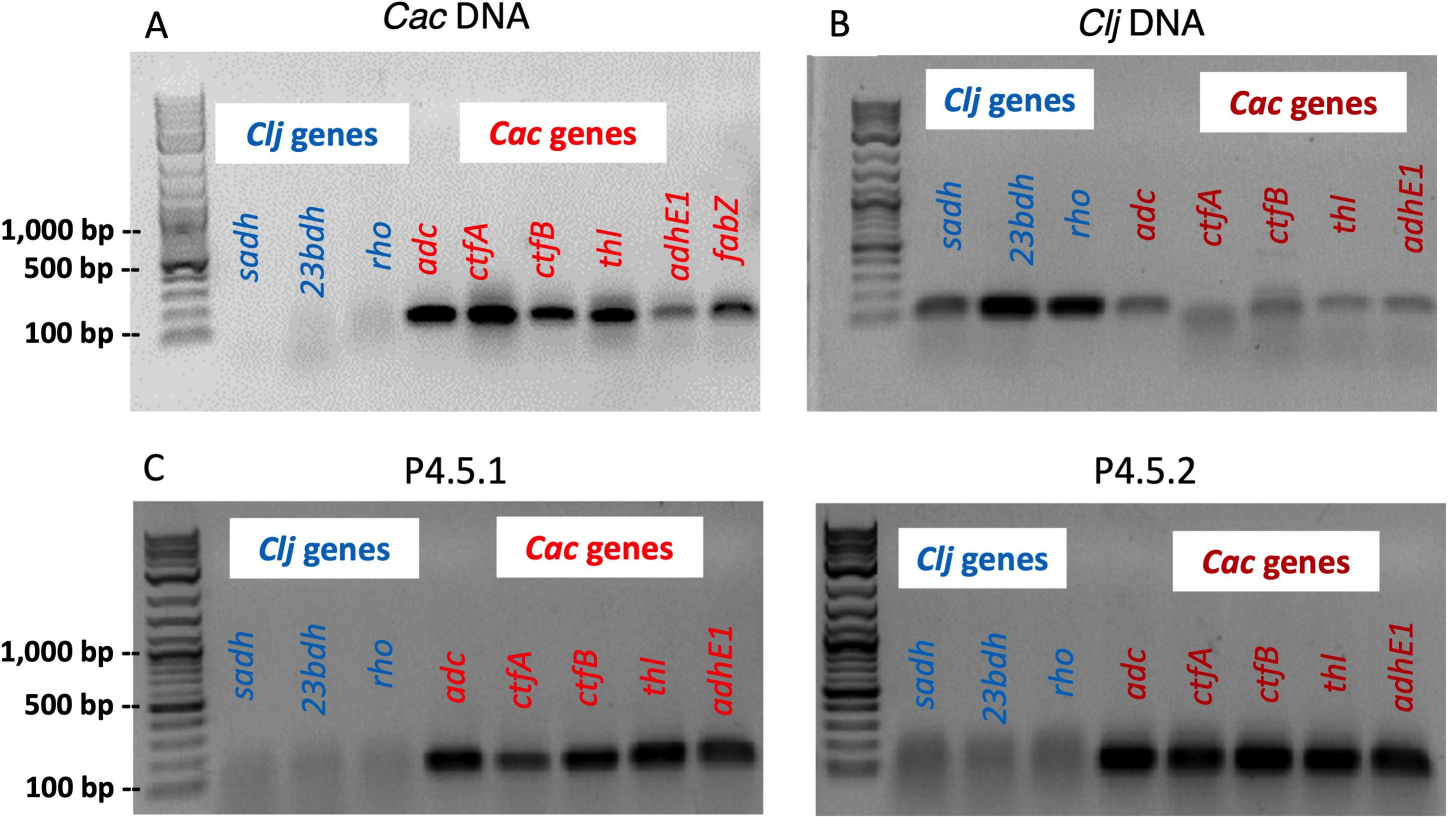
PCRs to test the presence of *C. acetobutylicum* and *C. ljungdahlii* characteristic genes in P4.5 cells. Three *C. ljungdahlii* (*Clj*) genes (*sadh, 23bdh,* and *rho*) and five or six *C. acetobutylicum* (*Cac*) genes (*adc, ctfA, ctfB, adhE1,* key solvent-formation genes encoded on the native pSOL1 megaplasmid*,* and *thl,* encoded on the main chromosome; panel A includes also the *fabZ* gene) were targeted. (**A**) *C. acetobutylicum*-specific genes generated the expected ~100 bp bands from the *C. acetobutylicum* genomic DNA template, while the *C. ljungdahlii-*specific primers did not. (**B**) *C. ljungdahlii-*specific genes generated the expected ~100 bp bands from the *C. ljungdahlii* genomic DNA template, while the *C. acetobutylicum* primers produced weak bands from non-specific binding due to the high A+T content of the two genomes. This is less important as the key test is the presence of *C. ljungdahlii* genes on DNA from PtP4.5 colonies in panel C. (**C**) PCR assays used to test genomic DNA of P4.5 cells for three *C. ljungdahlii*-specific and five *C. acetobutylicum-*specific genes. Results are shown for cells (P4.5.1 and P.4.5.2) derived from two PtP4.5 colonies.

The total DNA of P4.5 cells grown in liquid culture from individual PtP4.5 colonies was tested for five unique *C. acetobutylicum* and three unique *C. ljungdahlii* genes (21). Control PCRs with WT *C. acetobutylicum* genomic DNA produced bands (~100 bp) only with the *C. acetobutylicum*-specific primers (Fig. 3A). Similarly, control reactions with *C. ljungdahlii* genomic DNA produced strong bands (~100 bp) only with *C. ljungdahlii*-specific primers (Fig. 3B). PCR-assay results from P4.5 cells grown from two PtP4.5 colonies showed the presence of only *C. acetobutylicum* genes (Fig. 3C).

**Fig 4:**
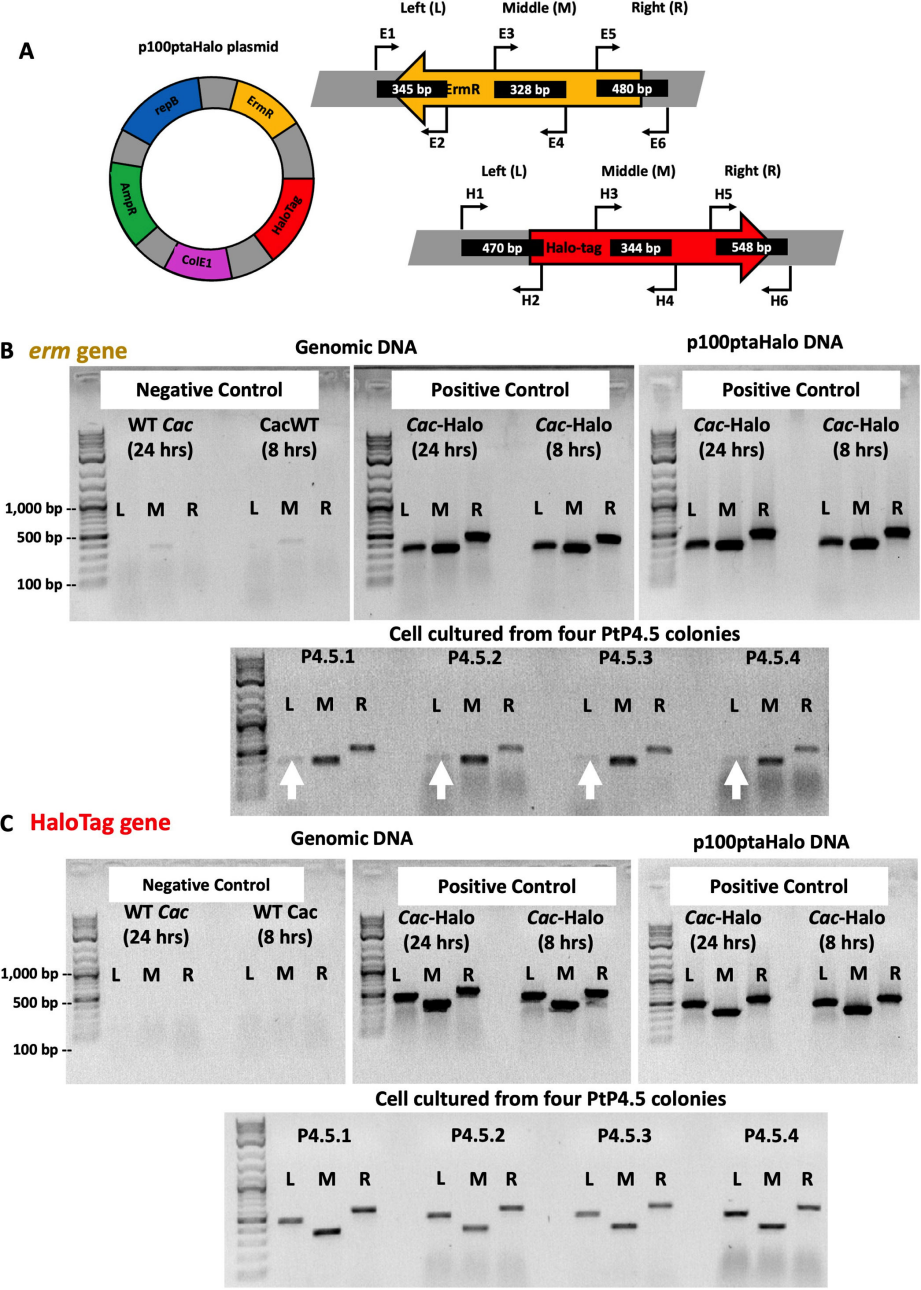
PCR assays to test the presence of the *erm* or HaloTag genes from the p100ptaHalo plasmid in P4.5 cells. (**A**) Three primer pairs were designed to bind to the left (L), middle (M), or right (R) of the *erm* and HaloTag genes on the p100ptaHalo plasmid. Primers E1 and H1 bind outside of each gene to the left, while primers E6 and H6 bind outside of each gene to the right, to test whether the original plasmid backbone was still present in the tested cells. For both genes, negative control used genomic DNA from WT *C. acetobutylicum*; positive control was DNA from *C. acetobutylicum*-ptaHalo cells or isolated p100ptaHalo plasmid. Cells for negative and positive controls and isolation of the p100ptaHalo plasmid were harvested either during the exponential phase (8 h) or an early stationary phase (24 h). Tested P4.5 cells were cultured from four PtP4.5 colonies and were harvested at 24 h of culture. The same amount of the DNA template was used in all *erm* and Halo PCR assays. We tested two culture time points of the controls to make sure there were no gene re-arrangements or plasmid instability issues as cells progressed from exponential (8 h) to the stationary phase (24 h). (**B**) PCR assays to detect the *erm* gene. We note that all four tested cell samples did not generate sharp, strong L bands (white arrows). (**C**) PCR assays to test the presence of the HaloTag gene.

PCR assays were also performed on P4.5 total DNA to test the presence of the *erm* or HaloTag genes from p100ptaHalo with three primer sets (Fig. 4A). Note that the E1 primer of the left (L) and the E6 primer of the right (R) set of probes bind outside the left and right ends, respectively, of the *erm* gene to probe the location of the *erm* gene in its plasmid context. Control PCRs for the *erm* gene showed identical results for both total and p100ptaHalo DNA from a *C. acetobutylicum* strain carrying the p100ptaHalo plasmid (strain *C. acetobutylicum-*ptaHalo) (Fig. 4B). DNA from four P4.5 colonies (P4.5.1 to P4.5.4) was tested for the presence of the *erm* gene. PCRs using the middle (M) and right (R) primer sets generated the expected bands (Fig. 4B). The reaction using the left (L) set of primers generated very faint bands for all colonies (Fig. 4B). Since P4.5 cells were resistant to Erm, the *erm* gene sequence must be complete. A few P4.5 cells might contain the full plasmid, yet all efforts to isolate the p100ptaHalo plasmid from these cultures failed. This implies that at least a portion of p100ptaHalo was integrated into the genome in a way that has disrupted the binding of the E1 primer thus resulting in faint L bands. This will be supported by the PacBio sequencing data below.

The same P4.5 DNA samples were also tested for the presence of the HaloTag gene. Control PCRs for the HaloTag gene showed identical results for both total and p100ptaHalo DNA from the *C. acetobutylicum-*ptaHalo strain (Fig. 4C). All three PCRs (L, M, and R) of the HaloTag assay produced the expected bands in all four tested P4.5 colonies (Fig. 4C) although less strong than the bands of those of the positive controls. Why were P4.5 cells non-fluorescent (Fig. S2) when labeled with the HaloTag ligand if they did carry the HaloTag gene? It is possible that P4.5 cells contain fewer HaloTag gene copies compared to *C. acetobutylicum*-ptaHalo.

Analysis of PacBio sequencing data from a culture grown from one PtP4.5 colony identified six sequence reads that contained the *erm* gene or the HaloTag gene, three of which contained two full copies of the p100ptaHalo plasmid, with no *C. acetobutylicum* genomic DNA present. A full p100ptaHalo copy was identified inserted in chromosome position 2,734,720, with ~3,000 bp and ~6,000 bp on either end of the read containing *C. acetobutylicum* genomic DNA, while two and a half copies of the plasmid were inserted into chromosome position 671,974, with ~7,000 bp of the read containing *C. acetobutylicum* genomic DNA. Analysis of sequenced reads from P4.5 suggests that the plasmid is integrated into the chromosome at two loci. Integration of the plasmid into the genome, along with a small number of reads containing only plasmid DNA, supports the PCR analysis data, as well as the flow cytometric and microscopy data. The PacBio data suggest that there are very few copies of the *erm* and HaloTag genes present in P4.5, and this is consistent with the PCR data (Fig. 4) for the *erm* and HaloTag genes. In addition, very low levels of HaloTag protein would be present which explains the lack of signal in the presence of HaloTag ligand in both flow cytometric and microscopy data (Fig. S3). PacBio data were also used to successfully assemble, *de novo*, the complete *C. acetobutylicum* genome including the pSOL1 megaplasmid, thus supporting the phenotypic characteristics of P4.5 cells. No genomic *C. ljungdahlii* DNA could be reliably identified in P4.5 sequencing data.

To support these findings, we also cultured and sequenced cells from one colony from each of PtP4.3 and PtP4.4. P4.3 sequencing data included six sequence reads that contained at least 500 bp of p100ptaHalo, with at least one end of the read containing *C. acetobutylicum* genomic DNA (insertions at 525,552; 1,325,197; 1,823,332; 2,702,390; 2,779,170; and 3,413,786 on the *C. acetobutylicum* genome). Four reads from P4.4 contained at least 500 bp of p100ptaHalo with at least one end containing *C. acetobutylicum* DNA (insertions at 349,946; 780,069; 980,388; and 2,204,734). Furthermore, in contrast to P4.5, there were a few hundred reads containing *C. ljungdahlii* DNA in P4.3, but considerably fewer reads in P4.4. There were no reads with both *C. ljungdahlii* DNA and p100ptaHalo DNA, which suggests that the plasmid was not integrated into the *C. ljungdahlii* genome. Taken together, these data suggest a dynamic integration—under Erm pressure—of p100ptaHalo into the *C. acetobutylicum* genome at each subculture passage, as the loci of integration events changed and the number of integration events decreased with each passage. The presence of *C. ljungdahlii* DNA in P4.3 and P4.4 suggests that at earlier stages of lineage 4, DNA from both organisms co-existed.

In summary, p100ptaHalo originating from *C. ljungdahlii*-ptaHalo strain successfully transferred to *C. acetobutylicum* and integrated into *C. acetobutylicum*’s genome at P4.5. We confirmed that *C. ljungdahlii* does not contain native conjugation machinery or mobile genetic elements (MGEs) that could be responsible for plasmid transfer to *C. acetobutylicum* by searching the reference genome using online Mobile Element Finder and ICEFinder tools (23, 24). Importantly, the reference *C. ljungdahlii* genome does not contain any integrative and conjugative elements (ICE), indicating the DNA exchange was not mediated by traditional conjugation (17).

### P1.5 and P2.5 cells show evidence of cells containing chromosomal DNA from both *C. acetobutylicum* and *C. ljungdahlii*

Subculturing of the other two coculture lineages resulted in the isolation of colonies from PtP1.5 and PtP2.5 which exhibited a more complex and unexpected phenotype compared to the phenotype of P4.5 cells described above. Single colonies from plates PtP1.5 and PtP2.5 were cultured in the liquid selection medium for analysis. Their phenotype was not consistent with either pure *C. ljungdahlii* or pure *C. acetobutylicum* strains. Starting with the 3rd subculture (Fig. 1), colonies of lineages 1 and 2 could not survive the heat shock, as would be the case for *C. ljungdahlii*. The cells from these same colonies had *C. acetobutylicum-*like phenotypes, including growth on glucose only, plus butanol and acetone production (data for P1.5 cells are shown in Fig. S6). Furthermore, both were Erm resistant, and a fraction of the cells was HaloTag fluorescent (data for P1.5 cells are shown in Fig. S6). Most surprising was the production of high concentrations of isopropanol (~80 mM titers) by P1.5 cells (Fig. S6), which should only be possible in the coculture of *C. acetobutylicum* and *C. ljungdahlii* or if hybrid *C. acetobutylicum*/*C. ljungdahlii* cells persist (21). SEM analysis (Fig. 2C and D) shows aberrant cell morphologies: an ultrastructure distinct from either *C. acetobutylicum* or *C. ljungdahlii* cells (see also Fig. S5) and some non-physiologically long cells (compared to the very long P4.3 cells of Fig. S4). In the coculture, *C. acetobutylicum* and *C. ljungdahlii* cells were found to undergo heterologous cell-to-cell fusion, which facilitates the exchange of proteins and RNA (19). Based on these data, cells from these colonies should contain characteristic genes of both *C. acetobutylicum* and *C. ljungdahlii* cells.

**Fig 5:**
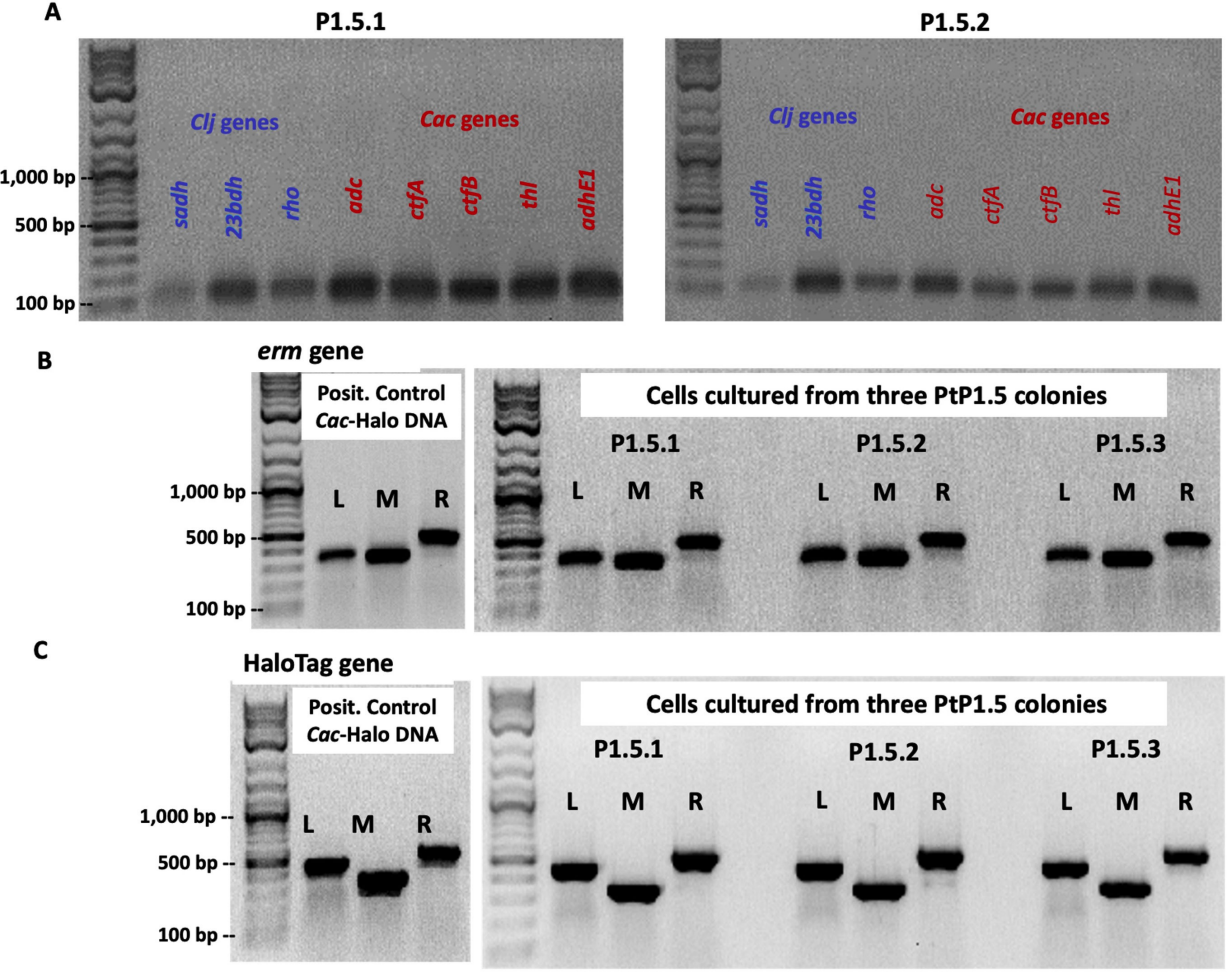
(**A**) PCR assays to test genomic DNA of cells grown from PtP1.5 colonies for three *C. ljungdahlii* (blue) and five *C. acetobutylicum* (red) genes. Results from two individual colonies are shown. Both were positive for the *C. ljungdahlii* (*Clj*) and *C. acetobutylicum* (*Cac*)-specific genes. (**B**) PCR assays used to detect the *erm* gene. DNA from the positive control produced the expected reaction products (bands), with strong intensity for the left (L), middle (M, and right (R) reactions. DNA extracts from three PtP1.5 colonies produced all three reaction products (bands); band intensities were similar to those of positive control. (**C**) PCR assays used to detect the HaloTag gene. DNA from the positive control produced the expected reaction products (bands), with strong intensity for the left (L), middle (M), and right (R) reactions. DNA from cells grown from three PtP1.5 colonies produced all expected reaction products (bands). Band intensity was similar to that observed in the positive control. The same amount of DNA template was used in each PCR. The left sub-panel of panel C (HaloTag gene) showing the positive control of *Cac*-Halo DNA is the same gel shown in Fig. 4C, left gel image under Genomic DNA, Positive Control, *Cac*-Halo (24 h).

Detailed PCR analysis was performed as above to test for the presence of *C. ljungdahlii* or *C. acetobutylicum* genes and the *erm* and HaloTag genes. In all tested cells grown from three individual colonies from a PtP1.5 plate, PCR data show that these cells carry all three *C. ljungdahlii* genes and all five *C. acetobutylicum* genes, as well as the full *erm* and HaloTag genes with PCR-product band intensities similar to those produced with genomic DNA from the *C. acetobutylicum*-ptaHalo strain (positive control) (Fig. 5). These data then suggest that P1.5 cells contain multiple copies of the *erm* and HaloTag genes, thus suggesting the presence of an intact p100ptaHalo plasmid. P1.5 cells contain a small population (1.3% to 8.0%) expressing the HaloTag protein at levels that result in a strong enough red signal to be detected by flow cytometry (Fig. S6). Therefore, at least some of the cells were able to maintain and express the p100ptaHalo plasmid over the course of the selection process. To confirm this, we were able to easily isolate the full p100ptaHalo plasmid from both P1.5 and P2.5 cells. PacBio sequencing data of cells grown from one colony from each of PtP1.5 and PtP2.5 were used to map the reads to the *C. ljungdahlii* and *C. acetobutylicum* reference genomes via PacBio SMRT Link analysis. This demonstrated that the full chromosome of each species (including the *C. acetobutylicum* pSOL1 native megaplasmid that carries all the genes for solvent formation) was present in P1.5 and P2.5 cells, as well as the full p100ptaHalo plasmid. In P1.5 cells, there were six reads with both p100ptaHalo DNA and *C. acetobutylicum* DNA detected. In P2.5 cells, there were 44 reads that contained both p100ptaHalo plasmid DNA and *C. acetobutylicum* DNA, and five reads that contained both p100ptaHalo DNA and *C. ljungdahlii* DNA. In addition, 259 reads were identified with >500 bp of p100ptaHalo plasmid from P1.5 and 7,016 reads from P2.5, which is consistent with the isolation of intact plasmid from each of these lineages.

### DNA methylation supports the existence of hybrid cells

The PacBio data can be used to examine the DNA methylation motifs of the two genomes in P1.5 and P2.5 cells aiming to support the presence of heterologous DNA in a single cell. We had collected DNA methylation data for *C. acetobutylicum* several years ago using PacBio sequencing data to examine the impact of metabolite stress (25, 26) on DNA methylation. The data were never published. We now show the experimentally determined methylation motifs in Table S1. We cannot find published experimentally determined DNA methylation motifs for *C. ljungdahlii,* but there are predictions in the REBASE database (27). Those are GATAAT/GTTAAT (abbreviated as GWTAAT, where W is A or T) and CAAAAAR. Thus, *C. ljungdahlii* and *C. acetobutylicum* share a major methylation motif: CAAAA^m6^AR. This motif is detected on both the *C. acetobutylicum* and *C. ljungdahlii* DNA from all PacBio data of this study.

#### Lineage 1, P1.5 cells

Both genomes are assembled as described above. On *C. acetobutylicum* DNA, the PacBio data detect the expected (Table S1) *C. acetobutylicum* motifs at high frequencies, but no *C. ljungdahlii* motifs. On *C. ljungdahlii* DNA, the PacBio data detect no *C. ljungdahlii* GWTAAT motif but they detect a few of two *C. acetobutylicum* motifs: GCDGC (the essential, canonical *C. acetobutylicum* DNA methylation motif G^m4^CDGCAGC/G^m4^CDGC) and CTTCAG/CTGAAG.

#### Lineage 2, P2.5 cells

On *C. acetobutylicum* DNA, the PacBio data detect the expected (Table S1) *C. acetobutylicum* motifs at high frequencies. But they also detected four (4) of the GWTAAT *C. ljungdahlii* methylation motif. On *C. ljungdahlii* DNA, in addition to the common CAAAAAR, the PacBio data detect strong methylation (about 50% of the theoretical possible) of the *C. ljungdahlii* GWTAAT motif, but no *C. acetobutylicum* methylation motifs.

These data suggest that the outcomes from lineages 1 and 2 are different. The methylation patterns of the *C. ljungdahlii* DNA of P1.5 cells suggest the coexistence of DNA from the two organisms in one cell, where the *C. acetobutylicum* methylation machinery dominates for some reason, possibly because of the faster replication of the *C. acetobutylicum* DNA. This clearly deserves further investigation.

### Clonal selection and estimation of DNA exchange frequency

As noted above, while colonies were selected from sparse plates, plating was not based on limiting dilution to ascertain strict clonality. Thus, it was possible that the colonies contained a mixed-cell population. To pursue this further and to collect quantitative colony data for estimating DNA transfer frequencies, we carried out three more successful sets of cocultures based on the schema of Fig. 1. All three generated distinct colonies upon selective plating using limiting dilution as described in Text S1. Data from one culture (which was followed most carefully quantitatively with respect to culture dilutions, sampling, and plating) was used to carry out a first estimation of DNA transfer frequencies (detailed in Text S1) as follows:

DNA transfer frequency based on donor cells = (31,200)/8.60 * 10^10^ = 3.63 * 10^−7^ (Eq. 1)

DNA transfer frequency based on recipient cells = (31,200)/2.1 * 10^10^ = 1.49 * 10^−6^ (Eq. 2)

## DISCUSSION

The goal of this study was to examine the possibility of DNA transfer between *C. ljungdahlii*-ptaHalo and WT *C. acetobutylicum* cells in coculture. Thus, a selection process was designed to identify any *C. acetobutylicum* that acquired plasmid DNA from *C. ljungdahlii*-ptaHalo. The selection process generated cells with unique phenotypes. First, P4.5 cells from PtP4.5 colonies had the expected phenotype of *C. acetobutylicum* cells (heat-shock survival, butanol and acetone production, no isopropanol nor 2,3-butanediol production, and expected TEM morphology) carrying the *erm* and HaloTag genes from the p100ptaHalo plasmid of the *C. ljungdahlii*-ptaHalo strain. Furthermore, p100ptaHalo plasmid DNA appears to have been integrated into the P4.5 genome. The interpretation of the PCR data of Fig. 4 is that the *erm* gene is integrated in chromosomal locations in a way that the E1 primer of the L set cannot always generate a good PCR product. If the p100ptaHalo plasmid had intact and had maintained episomally, all three sets (L, M, and R) would have produced strong bands. The PacBio data showed that the whole plasmid integrated into two chromosomal locations (and this would enable the E1 probe to bind), with additional integrations of the *erm* gene without all of the rest of the plasmid. These latter integrations were apparently such that the E1 primer could not bind to generate a PCR product. Thus, the faint bands from the cells of the PtP4.5 colonies represent products from the *erm* gene integrated differently in different locations of the chromosome, some enabling E1 binding, some not.

Regarding the PCR products of the Halo gene (Fig. 4), the less strong bands compared to positive controls suggest that the plasmid does not exist episomally in the P4.5 cells, because if it did, the bands would have been as strong as those of the control. This was verified by the PacBio data and the inability to isolate the p100ptaHalo plasmid from these cells. The PacBio data show that a few copies of HaloTag had integrated into the chromosome. Because the total template DNA was constant, all PCR conditions were identical, and the amount of PCR product was proportional to the HaloTag gene content of the template DNA, apparently then, the total HaloTag gene dosage was less than the dosage in control cells containing the episomally maintained plasmid. As a result, there is not a sufficiently high level of HaloTag protein for detectable fluorescence.

As discussed, there were relatively few reads in P4.5 cells that show p100ptaHalo-DNA integration, given the genome coverage of the sequencing data. The low read numbers are likely due to the standard protocol employed in our genomic facility for PacBio sequencing whereby only reads >6 kb were selected for sequencing, but it is still not clear why larger DNA reads containing p100ptaHalo DNA were not generated and sequenced. Nevertheless, given these data in combination with the PCR data of Fig. 4, the PacBio data of P4.3 and P4.5, and the strong phenotypic data of P4.5 cells, there is no question that P4.5 cells and earlier cells of the 4th lineage have acquired the p100ptaHalo plasmid and have integrated its DNA into the chromosome. PacBio-read numbers with mixed plasmid, and genomic *C. acetobutylicum* and *C. ljungdahlii* DNA were higher in P1.5 and P2.5 cells. Taken together, the data suggest complex DNA rearrangements giving rise to mosaic DNA and possibly a mixed cell population with dynamic DNA re-arrangements despite the use of single, distinct colonies. However, it should be emphasized that while colonies were selected from sparse plates, plating was not based on limiting dilution to ascertain strict clonality. While limiting dilution plating for the experiments to estimate DNA transfer frequencies provided strong evidence for hybrid cells, those cells will need to be characterized in detail. Nevertheless, the phenotypic, PCR, and PacBio sequencing data of P4.5 cells and the DNA methylation analysis clearly demonstrate interspecies DNA transfer and chromosomal integrations.

An interesting observation was that all lineages of Fig. 1 demonstrated some form of *C. acetobutylicum* resistance to Erm as *C. acetobutylicum* survived the presence of high concentrations of Erm from the very first subculturing passage of all lineages. One possibility would be an immediate transfer of the p100ptaHalo plasmid to WT *C. acetobutylicum* cells. Another possibility is the immediate formation of hybrid cells carrying both chromosomes and the p100ptaHalo plasmid, and there is evidence for such cells even in primary parent cultures (Fig. 8 of reference 19). However, we could not have anticipated that such hybrid cells would persist on the subculture and selection conditions of the present study. Yet, another possibility is the transient transfer of the Erm protein or mRNA from the *C. ljungdahlii*-ptaHalo to WT *C. acetobutylicum* cells, as part of the massive exchange of proteins and RNA between these organisms under co-culture conditions (19). It is not clear however how such transient acquisition of the Erm protein or mRNA would have persisted in subsequent selective subcultures.

The exchange and integration of plasmid DNA in the coculture is the first evidence of a novel HGT mechanism. Control transformation experiments showed that the restriction modification systems of *C. acetobutylicum* and *C. ljungdahlii* are incompatible (28), making the DNA transfer through a transformation route impossible. As neither species possesses a known Type IV or Type VI secretion system, transfer of DNA via these mechanisms is not possible. Chromosomal and plasmid DNA transfer via EVs is somewhat well documented in Gram^−^ cells, minimally so in pathogenic Gram^+^ cells (7, 8), but not in *Clostridium* organisms. In the well-documented case (29) in the Gram^+^ *Streptococcus mutans,* EVs are readily visible on the cell surface via SEM and are associated with profusely abundant eDNA nanowires visualized via SEM. None of these SEM-visible characteristics have been observed in our SEM images of monocultures or cocultures of *C. acetobutylicum* or *C. ljungdahlii*.

There is also no evidence of transduction via prophage excision and transfer of DNA. The *C. ljungdahlii* genome codes for a large 51 kb prophage and three more prophages scored as questionable or incomplete by the PHASTER tool (30, 31). *C. acetobutylicum* also has a 65 kb complete prophage and a 9 kb incomplete prophage (30, 31). If prophage excision was responsible for the transfer of plasmid DNA, we would have expected that the plasmid would have first integrated into the *C. ljungdahlii* chromosome near a prophage region and would have then been excised and transferred to *C. acetobutylicum* along with prophage genes. No *C. ljungdahlii* DNA was detected in P4.5 cells. Furthermore, there is no evidence of a traditional (versus distributive [17, 18]) conjugative machinery in either *C. acetobutylicum* or *C. ljungdahlii*. Distributive conjugal transfer (DCT) (17, 18) is an intraspecies (i.e., among strains of the same species) DNA transfer mechanism first demonstrated in mycobacteria. It is mediated through what is now known as a type VII secretion system (originally known as ESAT-6/WXG100) and leads to meiotic-like genome mosaicism, whereby multiple segments of donor DNA are co-transferred and integrated randomly into the recipient chromosome. In some way, the outcome of DCT seems to resemble the DNA transfer and integration reported here. DCT-mediated intraspecies DNA transfer requires extended (>18 h direct cell-to-cell contact on solid media or biofilms and does not occur in planktonic cultures (17). By contrast, the DNA transfer reported here is between different species and, as discussed above, it seems to take place in planktonic cultures. Nevertheless, the two mechanisms may share some structural and/or signaling components. The mechanism that initiates DCT upon cell-to-cell contact is not known, but models for how the DNA is transferred through pores generated by proteins of type VII secretion systems have been proposed (32). Diverse type VII secretion systems have been now identified in several organisms including in *Bacillus subtilis*, notably (33). Putative type VII genes for *C. acetobutylicum* have been computationally identified (34) and more homologous genes can be identified based on the *B. subtilis* type VII system (33), but none so far for *C. ljungdahlii*. However, in mycobacteria at least, a type VII secretion system is essential for DCT in the recipient but not the donor cells (35).

Thus, the observed exchange of DNA in the coculture was most likely facilitated through cell-to-cell fusion events that occur in *C. acetobutylicum–C. ljungdahlii* cocultures (19). Cell fusion between these two organisms was found to facilitate a large-scale exchange of protein and RNA (19), and, thus, it appears that plasmid-DNA exchange also took place during heterologous cell fusion. The exchange of protein and RNA may allow some plasmid DNA to escape the restriction modification of the recipient organism. To provide a definitive understanding of the events that took place and led to the observed phenotypes, one needs to visualize the presence of chromosomal and plasmid DNA from the two parent cells in single cells. This will require the development of new bioimaging technology not currently available for prokaryotes, such as the DNA PAINT technology (36–38). Genetic studies might also elucidate the molecular mechanism of heterologous cell fusion which will allow a better understanding of the mechanism of protein, RNA, and DNA transfer in these cocultures.

Beyond the transfer of plasmid DNA, heterologous fusion would also facilitate the exchange of mobile genetic elements, which are integrated into most prokaryotic genomes and can dynamically excise and reinsert (39, 40). The *C. acetobutylicum* genome does contain an IS1595-like insertion sequence, which encodes a transposase (CA_RS07810). *C. acetobutylicum* ATCC 824 encodes seven additional transposase genes (locus tags CA_RS01390, CA_RS03555, CA_RS03565, CA_RS03880, CA_RS08330, CA_RS13010, and CA_RS18140). While all but one of the transposases either contain premature stop codons or encode only partial proteins, one or more of these transposases could be responsible for a dynamic insertion and excision of the p100ptaHalo plasmid into the *C. acetobutylicum* genome. Evidence for movement of the plasmid or portions of the plasmid is seen in the PacBio data, as the plasmid is inserted in different locations in the genome in different generations of the lineage 4. Thus, heterologous fusion events may lead to novel cellular structures and phenotypes, which may lead to novel evolutionary trajectories in prokaryotic biology.

## MATERIALS AND METHODS

Information and requests for resources and reagents should be directed to and will be fulfilled by the corresponding author. All unique/stable reagents generated in the present study are available from the corresponding author with a completed materials transfer agreement.

### Microorganisms and culture media

Monocultures of *C. acetobutylicum* (ATCC 824), of the fluorescent strains of *C. ljungdahlii* strain (*C. ljungdahlii*-ptaHalo) and *C. acetobutylicum* (*C. acetobutylicum*-ptaHalo) (20), and their co-cultures, used the Turbo CGM medium, as described (19, 21). Briefly, Turbo CGM used for *C. ljungdahlii*-ptaHalo mono-cultures was supplemented with 5 g/L fructose (and for the recombinant strains with erythromycin [Erm; 100 µg/mL]), and cultures were grown in sealed bottles with 20 psig of H_2_/CO_2_ gas mixture (80/20%). Turbo CGM used for *C. acetobutylicum*-strain mono-cultures and co-cultures was supplemented with 5 g/L fructose and 80 g/L glucose, and cultures were grown in unsealed glass bottles in an anaerobic chamber (19, 21).

### Monoculture preparation and growth

WT *C. acetobutylicum* frozen stocks were streaked onto 2× YTG plates and cultured in Turbo CGM to generate seed cultures (21). *C. ljungdahlii*-ptaHalo and *C. acetobutylicum*-ptaHalo frozen stocks were inoculated into liquid Turbo CGM and passaged as needed to generate seed cultures and were supplemented with Erm (100 µg/mL) to maintain the plasmid DNA. The culture pH was adjusted to 5.2 after 12 h of growth with sterile deoxygenated 1 M NaOH to prevent acid death, as needed in *C. acetobutylicum* mono-cultures (21).

### Coculture setup

Cocultures of *C. acetobutylicum* and *C. ljungdahlii*-ptaHalo were prepared as reported (19, 21). Briefly, 5 mL of exponentially growing *C. acetobutylicum* seed cultures (OD_600_ of 1.0–2.0) were mixed with 90 mL of exponentially growing *C. ljungdahlii*-ptaHalo seed cultures (OD_600_ of 0.4–0.6). The *C. ljungdahlii*-ptaHalo cells were spun down at 5,000 rpm and washed twice in the fresh Turbo CGM medium to remove any residual Erm, before using them for coculture setup. The cocultures used for the DNA transfer were prepared at the R of ~10, where R is the ratio of *C. ljungdahlii* cells to *C. acetobutylicum* cells at the start of the co-culture (21), to ensure an excess of the plasmid-carrying *C. ljungdahlii*-ptaHalo cells (21). Co-cultures were performed in unpressurized static 100 mL glass bottles in the anaerobic chamber, with a total liquid volume of 30 mL. The pH of each coculture was adjusted to 5.2 with the sterile and deoxygenated NaOH after ~12 h of growth to prevent acid death (21).

### Selection procedure

After 24 h of the initial (parent) coculture, samples were collected for selection to isolate any *C. acetobutylicum* cells that acquired the p100ptaHalo plasmid (carrying the HaloTag gene and the Erm resistance gene [*erm*]; see reference 20 for details) from strain *C. ljungdahlii*-ptaHalo. The selection was done in liquid medium and solid plates. The liquid selection medium was the Turbo CGM medium containing 80 g/L of glucose, no fructose, and 100 µg/mL of Erm. The liquid selection cultures were done in unsealed 100 mL bottles in the anaerobic chamber. The plate selection was done on 2× YTG plates, containing 5 g/L of glucose, no fructose, and 100 µg/mL of Erm. During the liquid and solid selection, the presence of only high glucose concentration (a *C. acetobutylicum* substrate), but no fructose (*C. ljungdahlii* substrate), was expected to enrich the original cocultures samples in *C. acetobutylicum* cells while eliminating *C. ljungdahlii*-ptaHalo over the course of the selection. The selection media also contained Erm (100 µg/mL) to eliminate WT *C. acetobutylicum* cells during the selection process, and over time isolate only *C. acetobutylicum* cells that acquired the plasmid p100ptaHalo DNA (or parts of it) in the coculture. To start the selection process, 15 mL of samples from each mother coculture was washed in Turbo CGM medium (80 g/L of glucose only, no fructose) and transferred to 20 mL of the liquid selection medium. The coculture samples were washed to remove any fructose left over from the coculture growth medium. This was the first selection passage P*X*.1 (Fig. 1). After 24 h of growth, 15 mL of samples from each P*X*.1 culture was collected, washed, and transferred to fresh 20 mL of the selection liquid medium (passage P*X*.2). After 72 h of incubation, 1 mL of samples from P*1*.2 and P2.2 cultures was streaked onto 2xYTG selection plates (PtP*X*.3) to begin isolating and testing single colonies. Cultures P3.2 and P4.2 were incubated for 44 h before 1 mL of samples was streaked on selection plates. The selection plates developed colonies after 2 days of incubation at 37°C, except the selection plate PtP3.3. Multiple colonies (8–10) were picked from each selection plate and were cultured in the liquid selection medium (80 g/L glucose, 100 µg/mL Erm). Half of the selected colonies were heat shocked at 80°C for 10 min (per standard *C. acetobutylicum* culture techniques) to check whether the colonies from each plate were able to sporulate. All colonies that grew in the liquid selection medium were streaked again on the 2× YTG selection plate (plate PtP*X*.4), and the process was repeated one more time (plate PtP*X*.5) to further enrich each clone. Colonies from plates PtP1.5, PtP2.5, and PtP4.5 were grown in the selection liquid medium to generate cells for microscopy, flow cytometry, HPLC, and PCR analysis.

### Transmission electron microscopy

Samples from cultures were collected after 24 h of growth, fixed in 2% glutaraldehyde and 2% paraformaldehyde in 0.1 M sodium cacodylate buffer (pH 7.4), and stored at 4°C until further processing. The TEM sample processing and imaging were performed as described (19).

### Confocal fluorescence microscopy

Samples were collected after 24 h of growth, and labeled with the HaloTag-specific Janelia Fluor646 red ligand as described (19, 20). Labeled cells were placed in Nunc Lab-Tek chamber slides coated with poly-L-lysine. Cells were incubated for 1 h to immobilize cells on the poly-L-lysine coating. After 1 h, the chamber was rinsed with PBS thrice to remove excess cells, as described (19, 20). Immobilized cells were imaged using a confocal Carl Zeiss LSM880 microscope. Each sample was imaged using a 63×/1.4 oil objective (19, 20).

### Flow cytometry and fluorescent labeling of cells

Samples from cultures were collected, and labeled with the HaloTag-specific Janelia Fluor 646 red ligand as described (19, 20) to determine whether the isolated cells still produced the HaloTag protein. Flow cytometry analysis was performed as described (19, 20). The relationship between OD_600_ and cell numbers (Text S1) for the two organisms was determined using a CytoFlex S (Beckman Coulter) flow cytometer.

### HPLC metabolite analysis

Cells were grown in a liquid selection medium (Turbo CGM, 80 g/L glucose, no fructose, 100 µg/mL Erm) for 40 h, with pH adjustments at 12 h to keep the pH above about 5.2, to determine the fermentation profile of the cultured cells. Samples were collected approximately every 10–12 h for HPLC analysis (21).

### Plasmid isolation from isolated clones

Plasmid isolation used the NucleoSpin Plasmid Mini Kit (Macherey-Nagel) according to the manufacturer’s protocol. To test for the presence of a complete p100ptaHalo plasmid, the resulting plasmid preparation from each tested sample was transformed into chemically competent NEB 5-alpha *Escherichia coli* cells per the standard transformation protocol. Following transformation, *E. coli* cells were incubated at 37°C for 1 h, after which 150 µL of each transformation was plated on LB plates supplemented with 100 µg of ampicillin (p100ptaHalo plasmid contained Amp^R^ marker for *E. coli* transformation). The LB plates were incubated at 37°C for 24 h to allow *E. coli* colonies to develop. Plasmid preparations from cultures initiated from colonies from PtP1.5 and PtP2.5 produced *E. coli* colonies, indicating that the complete plasmid DNA was present in those samples. Plasmid preparations from cultures initiated from PtP4.5 colonies did not produce any colonies, indicating the complete plasmid was not present in these cells.

### PCR analysis of genomic and plasmid DNA

Cells were inoculated from distinct colonies of various plates described in the results and cultured in a liquid selective medium until cells reached an optical density of 1.0–2.0. Harvested cells were used for DNA extraction. Genomic and plasmid DNA was extracted from cultures for PCR analysis using the DNeasy Blood & Tissue Kit (Qiagen, Germany), following the procedure for Gram^+^ bacteria (21). Plasmid DNA was extracted using the NucleoSpin Plasmid DNA Kit (Macherey-Nagel, Germany). The same amount of DNA template was used in each PCR.

Extracted DNA from P1.5 and P4.5 cells were first tested by PCR using primers that target five selected *C. acetobutylicum* genes (*adc*, *ctfa*, *ctfb*, *adhe*, and *thl*) and three *C. ljungdahlii* genes (*sadh*, *23bdh*, and *rho*) to determine whether pure *C. acetobutylicum* was isolated in each line during the selection process. Primers used to screen for the selected *C. acetobutylicum* and *C. ljungdahlii* genes have been reported (21). PCR was performed using the green 2× Taq polymerase master mix (Fisher, MA). Each reaction was performed under the following conditions: initial 5 min denaturation at 95°C; followed by 25 cycles of 30 sec denaturation at 95°C, 30 s annealing at 65°C, and 30 s extension at 72°C; finished with 5 min extension at 72°C. All primer sets were designed to have the same annealing temperature of 65°C.

Genomic and plasmid DNA samples from P1.5 and P4.5 cells were also tested for the presence of the HaloTag, and erythromycin resistance (*erm*) genes. Each individual colony selected for the assay was grown in the liquid selection medium until they reached an optical density of 1.0–2.0. Cells collected from each culture were used for genomic DNA extraction. Three primer sets were designed for each gene, where the left pair (L) spanned the 5′-end of the gene and the plasmid backbone DNA located to the left of the gene, the middle pair (M) spanned a region of each gene in the middle, and the right pair (R) spanned the 3′-end of the gene and the plasmid backbone DNA located to the right. This is summarized visually in Fig. 4. Primer sequences are shown in Table S2. PCR was performed using the green 2× Taq polymerase master mix (Fisher, MA). Each reaction for the *erm* gene (L, M, and R) was performed under the following conditions: initial 5 min denaturation at 95°C; followed by 25 cycles of 30 s denaturation at 95°C, 30 s annealing at 53°C, and 35 s extension at 72°C; finished with 5 min extension at 72°C. Each reaction for the HaloTag gene (L, M, and R) was performed under the same conditions as above, except the annealing temperature was 53°C for these primer pairs (Table S2). 5 µL of each amplification reaction was run on an agarose gel. Gels were imaged using the ChemiDoc XRS+ Gel Imaging System (Biorad).

### Whole-genome SMRT PacBio Sequencing and Bioinformatics

Isolated colonies were cultured overnight in TCGM medium with Erm and high molecular weight (HMW) DNA was isolated using the MagAttract HMW DNA Kit (Qiagen) according to the manufacturer’s instructions. Single-molecule real-time sequencing was performed at the University of Delaware DNA Sequencing and Genotyping Center. SMRTbell DNA libraries were constructed as described (41). Libraries were size-selected starting at 6 kb with an average library size of 10 kb, as measured by Fragment Analyzer (Advanced Analytical Technologies, Inc.). DNA Sequencing was performed on PacBio Sequel II Single-Molecule Sequencer (Pacific Biosciences, Menlo Park, CA) instrument using P4-C2 chemistry, mag-bead loading, and 3 h movie time. Reads from each sample were assembled into contigs using SMRT Analysis version 10.1 through the SMRT Portal. The CCS (Circular Consensus Sequence) tool from PacBio SMRT Tools v10.1 was used to calculate the consensus sequences from the subreads in the PacBio data for each sample. DNA methylation analysis was carried out as described (41). The CCS reads were then aligned to the *erm* gene, HaloTag gene, and the entire p100ptaHalo plasmid using BLASTn (v2.11.0) (42). Reads aligning to more than 500 contiguous base pairs of either the *erm* gene, HaloTag gene, or the plasmid were then mapped to the *C. acetobutylicum* reference genome (GCF_000008765.1) to identify possible integration sites using minimap2 (v2.1) (43). These reads were also aligned to the *C. acetobutylicum* reference genome using BLASTn to filter by alignment length and percent identity. In addition, the presence of *C. ljungdahlii* DNA in each sample was examined by mapping the reads to the *C. ljungdahlii* reference genome (GCF_000143685.1) and identifying which reads mapped to *C. ljungdahlii* better than *C. acetobutylicum* and to *C. ljungdahlii* only. The presence of *C. ljungdahlii* DNA with plasmid was examined by aligning the PacBio reads that contained more than 500 contiguous base pairs of plasmid to the *C. ljungdahlii* reference genome (GCF_000143685.1) using BLASTn. Online tools ICEFinder, Mobile Element Finder, and PHASTER were used to search the reference genomes of *C. acetobutylicum* ATCC 824 and *C. ljungdahlii* DSM 13528 genomes for integrative and conjugative elements, mobile genetic elements and prophages, respectively (23, 24, 30, 31).

## Data Availability

Sequencing data has been deposited in NCBI Sequence Read Archive with corresponding BioSample accession numbers SAMN24718981, SAMN24718982, and SAMN24718983 under the project accession number PRJNA795347.

## SUPPLEMENTAL MATERIAL

Text S1 mbio.03133-23-s0001.docx Calculation of DNA transfer frequencies using clonal plating.

Figure S1 mbio.03133-23-s0002.docx Cell densities.

Figure S2 mbio.03133-23-s0003.docx Metabolite profile of the cells grown from colonies from PtP4.5 plates.

Figure S3 mbio.03133-23-s0004.docx Flow cytometric analysis of cells grown from isolated colonies from plates PtP4.3, PtP4.4, and PtP4.5.

Figure S4 mbio.03133-23-s0005.docx Confocal fluorescence imaging of P4.3 cells.

Figure S5 mbio.03133-23-s0006.docx Comparison of morphologies of various strains and species.

Figure S6 mbio.03133-23-s0007.docx Metabolite profile of cells grown from two PtP1.5 colonies and flow cytometric analysis of the two cultures.

Figure S7 mbio.03133-23-s0008.docx Quantification of plasmid transfer efficiency.

Table S1 mbio.03133-23-s0009.docx DNA modification motifs in *Clostridium acetobutylicum* using published PacBio sequencing data.

Table S2 mbio.03133-23-s0010.docx Primer pairs used for the HaloTag and the erythromycin resistance (*erm*) gene PCR assays.

ASM does not own the copyrights to Supplemental Material that may be linked to, or accessed through, an article. The authors have granted ASM a non-exclusive, world-wide license to publish the Supplemental Material files. Please contact the corresponding author directly for reuse.
