# Supplementary material for: DNA transfer between two different species mediated by heterologous cell fusion in Clostridium coculture
Source: mBio. 2024 Jan 12;15(2):e03133-23. doi: 10.1128/mbio.03133-23 (PMC10865971; doi:10.1128/mbio.03133-23)
Supplement: Text S1 — Calculation of DNA transfer frequencies using clonal plating. [file mbio.03133-23-s0001.docx]

**Supplementary Text 1**

**Text S1. Calculation of DNA transfer frequencies using clonal plating**

To estimate the frequency of DNA transfers that took place during the seminal coculture (CC), we carried out three cocultures according to the schema of Fig. 1 up to the plating following the 2^nd^ subculture but using limiting dilution plating to ensure clonality (Fig. S7). We counted cells that give rise to sprawling, flower (or star) shaped colonies on selective glucose/erythromycin plates after the 2^nd^ selective subculture P2. *C. ljungdahlii*-ptaHALO forms small, translucent, circular colonies which are easily distinguishable and grow slower than *C. acetobutylicum* strains with erythromycin resistance. Since erythromycin is a bacteriostat, the two selective cultures (totaling over 72 hours) were needed to fully quench any residual *C. acetobutylicum* metabolism, which could sustain *C. ljungdahlii* by the production of H_2_ and CO_2_, as evidenced by the gradual rise in optical density in P1 (from OD_600_ 6.5 to 9; Fig. S7.A). It is not possible to estimate the growth during P1 of *C. acetobutylicum* cells that acquired erythromycin resistance, but this should be small and, thus, the estimation of DNA transfer is not significantly affected.

First, we determined the concentration of CFU/mL in the second selection P2 culture (Fig. S7.B):

39 CFU / 66.7 uL of culture = 585 CFU/mL of culture

Next, we calculated the total number of CFUs in the second selection P2 culture:

585 CFU/mL * 20 mL (volume of the second selection culture) = 11,700 CFU in P2.

Next, we accounted for the two passages (P1, P2) which are effectively dilutions:

15 mL out of 30 mL passaged from seminal CC to P1  = 2x dilution

15 mL out of 20 mL passaged from P1 to P2                 = 1.33x dilution

Thus, the number of *C. acetobutylicum* or hybrid cells that have acquired the erythromycin resistance during the seminal CC is:

11,700 CFU * 2 *1.33 = 31,200 CFU.

On the basis of initial donor (*C. ljungdahlii*) or recipient (*C. acetobutylicum*) cells, the frequency of DNA exchange is:

DNA transfer frequency based on donor cells = (31,200)/ 8.60*10^10^ = 3.63*10^-7^ (Eq. 1)

DNA transfer frequency based on recipient cells = (31,200) / 2.1*10^10^ = 1.49*10^-6^ (Eq. 2)

Here the number of *C. ljungdahlii* or *C. acetobutylicum* in the culture inoculum was determined by the product of the pre-culture OD_600_, volume, and the correlation between OD_600_ and cell density. The correlation between 1 OD_600_ absorbance unit and cell density is 1.87x10^9^ and 3.00x10^9^ for *C. ljungdahlii* and *C. acetobutylicum,* respectively, which we determined by flow cytometry.

Similar but 15-20-fold lower frequencies were estimated from the other two biological replicates.

**Comments**. Plating of *C. acetobutylicum* like most clostridia is not very effective due to commitment to sporulation and thus these CFUs represent conservative cell numbers. It is also likely that not all “hybrid” cells form proper or large colonies, and for these calculations we only counted large colonies and not all colonies. The restriction modification system of *C. acetobutylicum* imposes an additional barrier to DNA transfer, as well. Note also that the more accurate flow-cytometry based estimation of cell number per unit OD_600_ is 5 times higher than previously reported numbers for *C. acetobutylicum* (6x10^8^ cells/OD_590_) (1) using manual cell counting. Reported *E. coli* numbers are 6 to 8x10^8^ cells/OD_600_ (2). **For these reasons, it is likely that the estimated DNA transfer frequencies above are conservative**.

***References***

1. Reardon K. 1988. PhD Dissertation (p 62) California Institute of Technology, Pasadena, California

2. Volkmer B, Heinemann M. 2011. Condition-dependent cell volume and concentration of Escherichia coli to facilitate data conversion for systems biology modeling. PLoS One 6:e23126.
