# Supplementary material for: DNA transfer between two different species mediated by heterologous cell fusion in Clostridium coculture
Source: mBio. 2024 Jan 12;15(2):e03133-23. doi: 10.1128/mbio.03133-23 (PMC10865971; doi:10.1128/mbio.03133-23)
Supplement: Figure S1 — Cell densities. [file mbio.03133-23-s0002.docx]

**Supplementary Figure S1**

**FIG. S1.** Cell densities of the starting co-cultures (A), 1^st^ liquid passage (B), and 2^nd^ liquid passage (C). The arrows in panel A indicate at which point timepoint the co-cultures were sampled to start the 1^st^ liquid passages (22 hrs for co-cultures 1.0 and 2.0 and 26.5 hrs for co-cultures 3.0 and 4.0). The 1^st^ liquid passages were cultured in the selection medium for 25 hrs before being passaged again. The 2^nd^ liquid passages were cultured for 72 hrs (passages P1.2 and P2.2) or for 44 hrs (passages P3.2 and P4.2). OD is OD_600_.
