# Supplementary material for: DNA transfer between two different species mediated by heterologous cell fusion in Clostridium coculture
Source: mBio. 2024 Jan 12;15(2):e03133-23. doi: 10.1128/mbio.03133-23 (PMC10865971; doi:10.1128/mbio.03133-23)
Supplement: Figure S2 — Metabolite profile of the cells grown from colonies from PtP4.5 plates. [file mbio.03133-23-s0003.docx]

**Supplementary Figure 2**
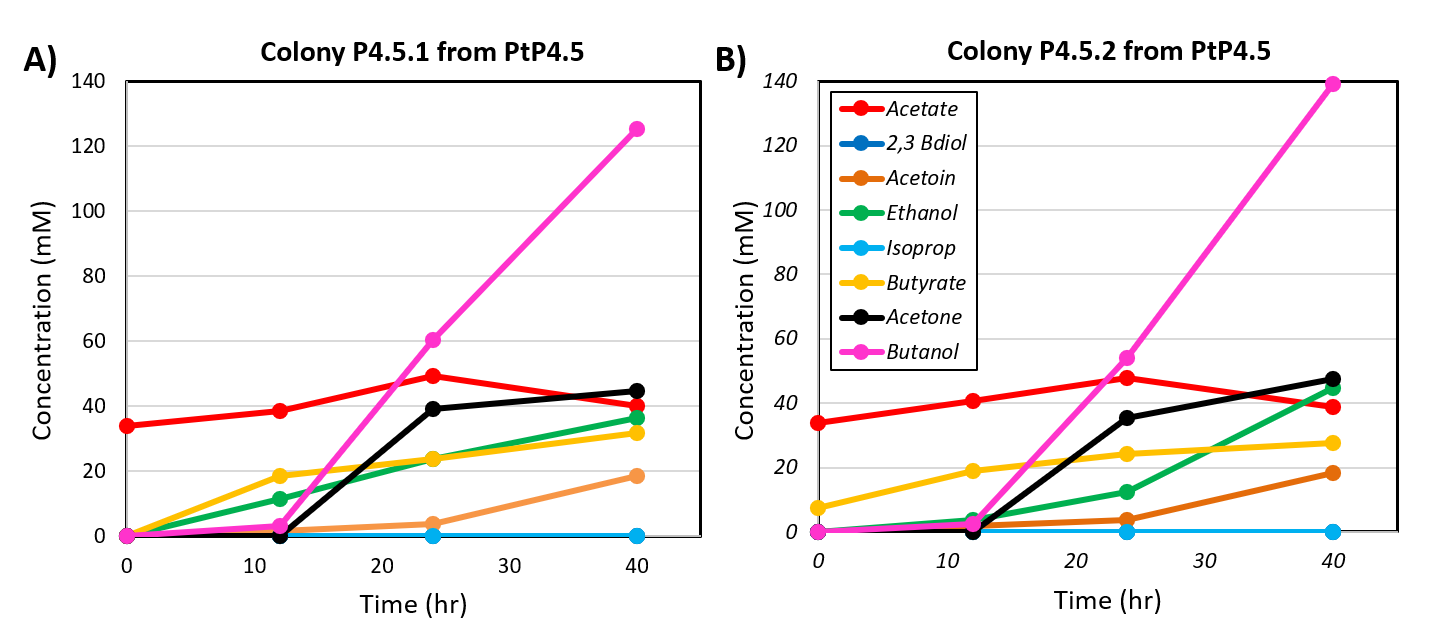


**FIG. S2.** Metabolite profile of the cells grown from colonies from PtP4.5 plates. Panels (A) and (B) show results for cultures from two colonies cultured in selective liquid medium (glucose, Erm, no fructose).
