# Supplementary material for: DNA transfer between two different species mediated by heterologous cell fusion in Clostridium coculture
Source: mBio. 2024 Jan 12;15(2):e03133-23. doi: 10.1128/mbio.03133-23 (PMC10865971; doi:10.1128/mbio.03133-23)
Supplement: Figure S3 — Flow cytometric analysis of cells grown from isolated colonies from plates PtP4.3, PtP4.4, and PtP4.5. [file mbio.03133-23-s0004.docx]

**Supplementary Figure 3**

**FIG. S3.** Flow cytometric analysis of cells grown from isolated colonies from plates PtP4.3, PtP4.4, and PtP4.5. Cells were labeled with the red Janelia Fluor®646 ligand. Data each from two independent cultures are shown (e.g., P4.4.1 and P4.4.2).
