# Supplementary material for: DNA transfer between two different species mediated by heterologous cell fusion in Clostridium coculture
Source: mBio. 2024 Jan 12;15(2):e03133-23. doi: 10.1128/mbio.03133-23 (PMC10865971; doi:10.1128/mbio.03133-23)
Supplement: Figure S4 — Confocal fluorescence imaging of P4.3 cells. [file mbio.03133-23-s0005.docx]

**Supplementary Figure 4**


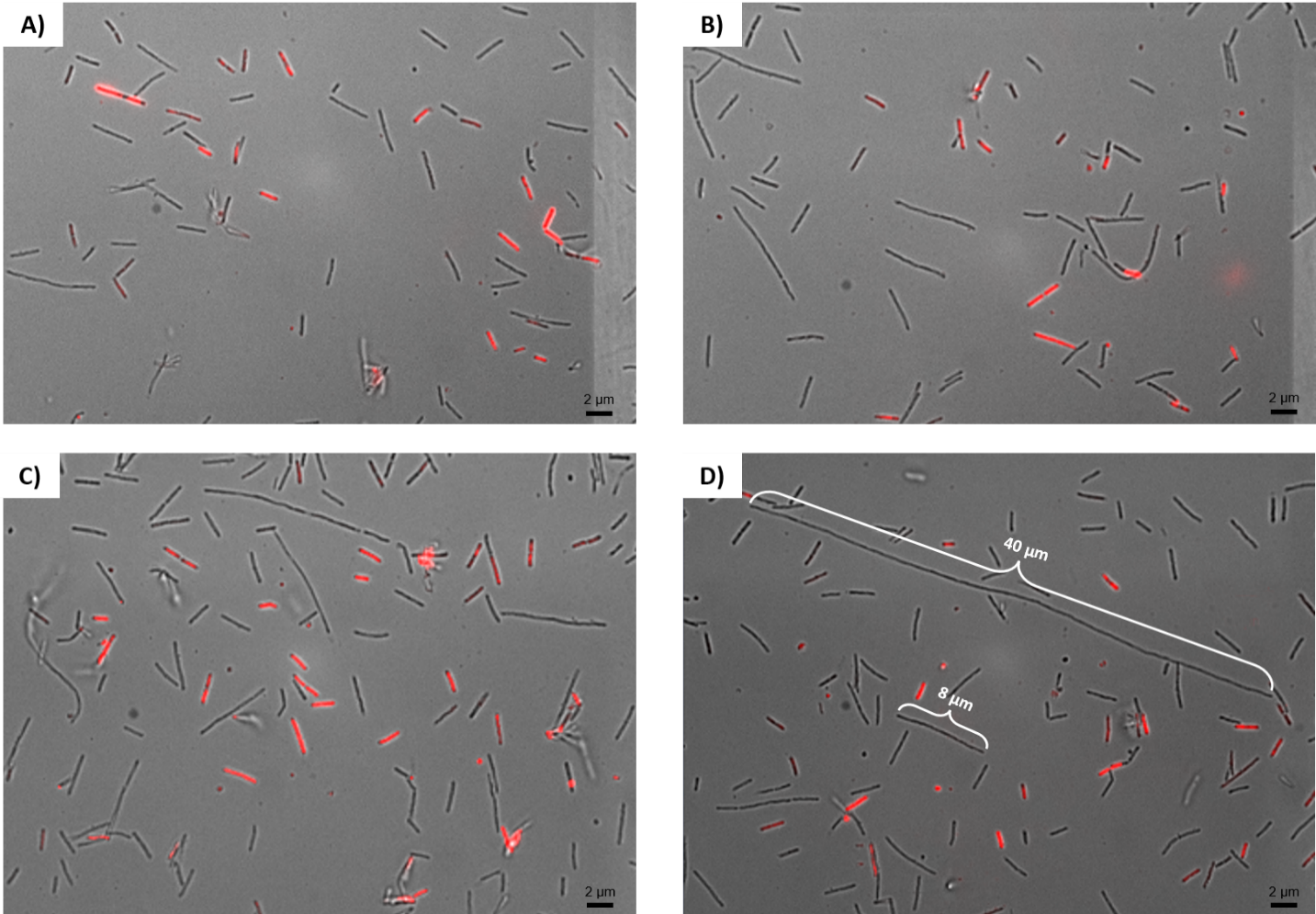


**FIG. S4.** Confocal fluorescence imaging of P4.3 cells. Cells were labeled with the red Janelia Fluor®646 ligand (A-D). Panel (D) shows elongated cells ranging from 8 μm up to 40 μm in length, none with red fluorescence. 2 μm bar at lower right.
