# Supplementary material for: DNA transfer between two different species mediated by heterologous cell fusion in Clostridium coculture
Source: mBio. 2024 Jan 12;15(2):e03133-23. doi: 10.1128/mbio.03133-23 (PMC10865971; doi:10.1128/mbio.03133-23)
Supplement: Figure S5 — Comparison of morphologies of various strains and species. [file mbio.03133-23-s0006.docx]

**Supplementary Figure 5**

**FIG. S5.** Comparison of morphologies of various strains and species. WT *C. acetobutylicum* (*Cac*) exhibited large translucent regions within its cytoplasm called granulose, which form as part of its sporulation program. *C. ljungdahlii* (*Clj*) cells remain homogenously electron dark, as is typical of vegetative cells with no signs of any differentiation/sporulation. TEM imaging of P1.5 cells showed a mixed morphology unlike either *Cac*- or *Clj*-cell morphologies. TEM imaging of P4.5 cells were similar to those of WT *Cac* cells.
