# Supplementary material for: DNA transfer between two different species mediated by heterologous cell fusion in Clostridium coculture
Source: mBio. 2024 Jan 12;15(2):e03133-23. doi: 10.1128/mbio.03133-23 (PMC10865971; doi:10.1128/mbio.03133-23)
Supplement: Figure S6 — Metabolite profile of cells grown from two PtP1.5 colonies and flow cytometric analysis of the two cultures. [file mbio.03133-23-s0007.docx]

**Supplementary Figure 6**


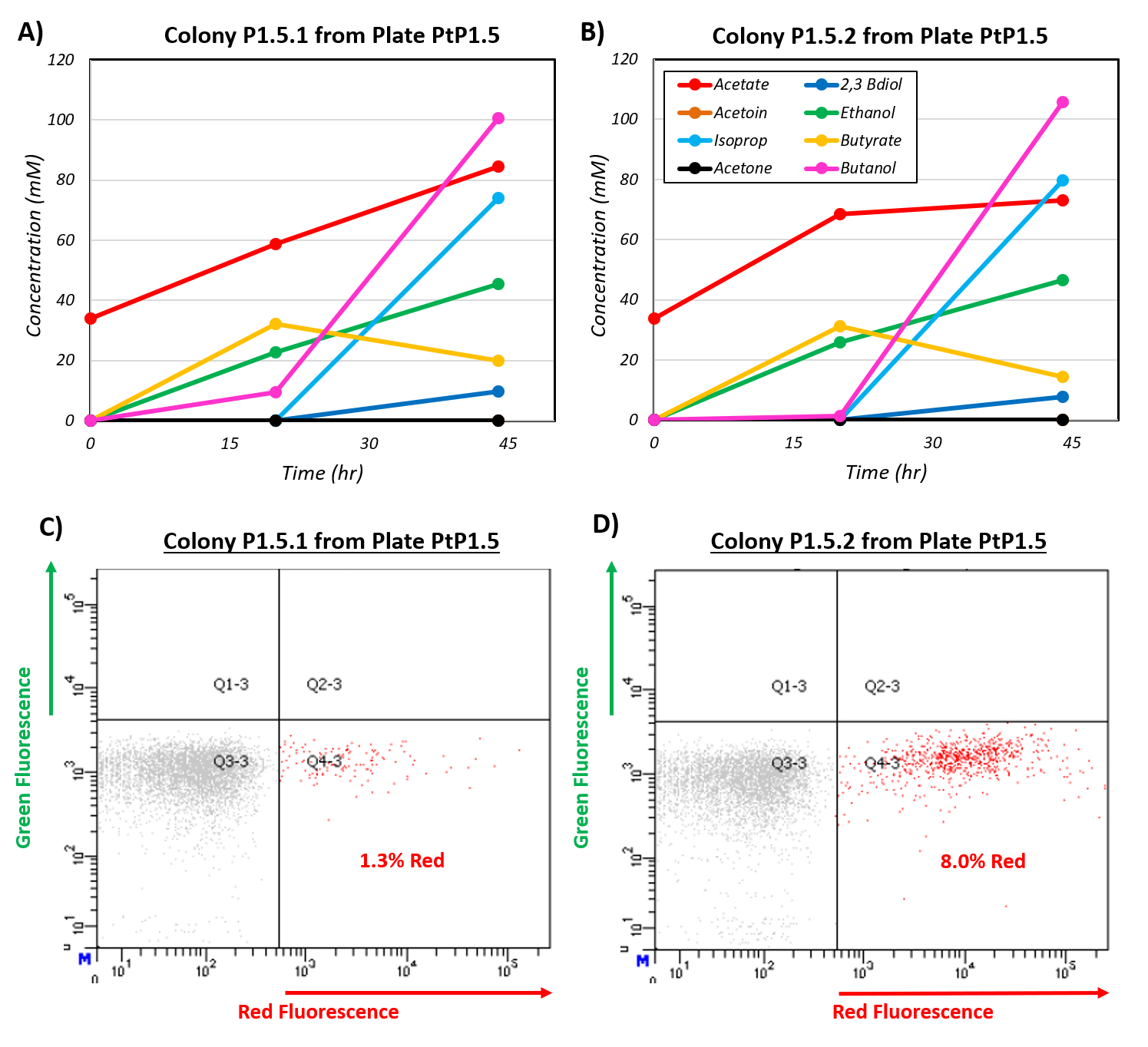


**FIG. S6.** (A) and (B): Metabolite profile of cells grown from two PtP1.5 colonies. Two individual colonies from plate PtP1.5 were picked and cultured in the liquid selective medium. The two resulting cultures were used for metabolite and fluorescence analysis. (C) and (D): Flow cytometric analysis of the two cultures. Cells were labeled with the red Janelia Fluor®646 ligand.
