# Supplementary material for: DNA transfer between two different species mediated by heterologous cell fusion in Clostridium coculture
Source: mBio. 2024 Jan 12;15(2):e03133-23. doi: 10.1128/mbio.03133-23 (PMC10865971; doi:10.1128/mbio.03133-23)
Supplement: Figure S7 — Quantification of plasmid transfer efficiency. [file mbio.03133-23-s0008.docx]

**Supplementary Figure 7**

**FIG. S7:** Quantification of plasmid transfer efficiency. **(A)** Optical density (OD_600_) of the seminal coculture (CC) and the subsequent selection cultures (P1, P2). After 20 hours of growth, the seminal CC (dark red) was passaged into the first selection culture (P1, blue). After 27 hours, the first selection culture P1 was passaged into the second selection culture (P2, green). After 44 hours, serial 10x dilutions were plated onto 2xYTG selection plates and incubated at 37°C for 4 days anaerobically on glucose, erythromycin plates. **(B)** Images of the serial dilution plates. From left to right, 10^0^ , 10^-1^, and 10^-2^. The 10^-2^ dilution represents clonal plating. The plated culture volume and CFU are indicated for each plate. 3 random flower/star-like colonies (B, C, and E) were inoculated into selection media (glucose, erythromycin). **(C)** The optical density (OD_600_) of the three randomly selected colonies from the plates of panel **(B)**. **(D)** Glucose consumption and metabolite concentrations at the end of the culture. The strong growth in liquid media and plates containing glucose and erythromycin, and the presence of 4 carbon metabolites (butyrate and butanol) and of isopropanol but no acetone indicate that *C. acetobutylicum* has acquired erythromycin resistance from *C. ljungdahlii*-ptaHALO through horizontal gene transfer, and that these colonies, in view of the clonal plating, represent hybrid cells, as *C. acetobutylicum* cannot produce isopropanol. TMTC: too many to count.
