# Supplementary material for: DNA transfer between two different species mediated by heterologous cell fusion in Clostridium coculture
Source: mBio. 2024 Jan 12;15(2):e03133-23. doi: 10.1128/mbio.03133-23 (PMC10865971; doi:10.1128/mbio.03133-23)
Supplement: Table S1 — DNA modification motifs in Clostridium acetobutylicum using published PacBio sequencing data. [file mbio.03133-23-s0009.docx]

**Supplementary Table 1**

**Table S1.** DNA modification motifs in *Clostridium acetobutylicum* using published PacBio sequencing data (1, 2). Boldface is to indicate the methylated base. R is A or G ; S is G or C; N is A, T, C, or G

| Motif | Modified position | Modificat. Type | % Motifs Detected* | # of Motifs Detected* | # of Motifs in Genome | Partner Motif |
| --- | --- | --- | --- | --- | --- | --- |
| CTGA**^m6^A**G | 5 | m6A | 99% | 2218 | 2236 | CTTCAG |
| CTTC**^m6^A**G | 5 | m6A | 99% | 2216 | 2236 | CTGAAG |
| CAAAA**^m6^A**R | 6 | m6A | 94% | 3730 | 3974 | YTTTTTG |
| G**^m6^A**STC | 2 | m6A | 76% | 2278 | 2996 | GASTC |
| G**^m4^C**NGC^¶^ | 2 | m4C | 6% | 870 | 14576 | GCNGC |
| G**^m4^C**NGCAGC | 2 | m4C | 52% | 226 | 437 | GCTGCNGC |

* Average of 6 samples (non-stressed, butyrate-stressed, butanol-stressed each with 2 biological replicates)(1, 2).

^¶^This motif is a sub-motif of G**^m4^C**NGCAGC

***References***

1. Venkataramanan KP, Jones SW, McCormick KP, Kunjeti SG, Ralston MT, Meyers BC, Papoutsakis ET. 2013. The *Clostridium* small RNome that responds to stress: the paradigm and importance of toxic metabolite stress in C*. acetobutylicum*. BMC Genomics 14:Article 847. doi: 10.1186/1471-2164-14-849.

2. Venkataramanan KP, Min L, Hou SY, Jones SW, Ralston MT, Lee KH, Papoutsakis ET. 2015. Complex and extensive post-transcriptional regulation revealed by integrative proteomic and transcriptomic analysis of metabolite stress response in *Clostridium acetobutylicum*. Biotechnology for Biofuels 8:Article 81. doi: 10.1186/s13068-015-0260-9.
