# Supplementary material for: DNA transfer between two different species mediated by heterologous cell fusion in Clostridium coculture
Source: mBio. 2024 Jan 12;15(2):e03133-23. doi: 10.1128/mbio.03133-23 (PMC10865971; doi:10.1128/mbio.03133-23)
Supplement: Table S2 — Primer pairs used for the HaloTag and the erythromycin resistance (erm) gene PCR assays. [file mbio.03133-23-s0010.docx]

**Supplementary Table 2**

**Table S2.** Primer pairs used for the HaloTag and the erythromycin resistance (*erm*) gene PCR assays. Primers were designed using Primer3Plus online tool (https://www.bioinformatics.nl/cgi-bin/primer3plus/primer3plus.cgi). The annealing temperature (T_A_), the expected product size, the directionality, and the sequence of each primer are listed.

| **Gene** | **Reaction** | **Size (bp)** | **For/Rev** | **Primer Sequence (5’ – 3’)** |
| --- | --- | --- | --- | --- |
| *erm* Gene  (T_A_=50°C) | Left  (L) | 345 | F | AGATACTGCACCCCCTGAAC |
|  |  |  | R | TTTCGTTATGAAATGGGTTAACAA |
|  | Middle  (M) | 328 | F | TAATGCCAATGAGCGTTTTG |
|  |  |  | R | TGAAATCGGCTCAGGAAAAG |
|  | Right  (R) | 480 | F | CTTTTCCTGAGCCGATTTCA |
|  |  |  | R | TATTTCACTTAGGCATTTCACG |
| HaloTag  Gene  (T_A_=53°C) | Left  (L) | 470 | F | GGTCGTAGAGCACACGGTTT |
|  |  |  | R | CGTAATGAGGGTCAAATGGG |
|  | Middle  (M) | 344 | F | CCCATTTGACCCTCATTACG |
|  |  |  | R | CTCTTTCAGGGTTTCTCTTTGC |
|  | Right  (R) | 548 | F | TTCTGAGATTGCAAGGTGGTT |
|  |  |  | R | TTTTTGTGATGCTCGTCAGG |
